# Analysis of the causes of N/P imbalance in mangrove water caused by high elevation shrimp ponds

**DOI:** 10.1038/s41598-025-02440-x

**Published:** 2025-05-20

**Authors:** Yunan Yang, Zhe Li, Nan Zhou, Yangang Lin, Qian Sheng, Myat Thiri, Yao Wang

**Affiliations:** 1 School of Space and Earth Sciences, Beihang University, Beijing, 100191 China; 2 Biotechnology Research Department, Ministry of Education, Kyauk Se Township, Mandalay Division, 15011 Myanmar

**Keywords:** Marine chemistry, Wetlands ecology

## Abstract

Due to its unique estuarine location at the junction of land and sea, mangrove wetlands are surrounded by numerous high-elevation shrimp ponds. The high-elevation shrimp ponds around the mangrove forest undergo 2.3 clearances by quicklime (CaO) disinfectant per year in China, but the impact of the quicklime disinfectant used and emitted on the mangrove wetland ecosystem is seriously underestimated. Due to the relatively limited data provided by high-elevation shrimp pond aquaculture in estuarine areas for the mangrove ecosystem, this study established an algorithm for calculating the reaction rate of quicklime disinfectants used in high-elevation shrimp pond aquaculture, which is the fundamental reason for the imbalance of N/P ratio in mangrove wetlands. Results showed that the amount of Ca(OH)_2_ produced by quicklime during the initial cleaning of the shrimp pond was 1303.4 t/a. The annual consumption of Ca(OH)_2_ by organic acids, strong chlorine disinfectants, and TP in the marine system was 154.6–171.5 t, 1.7 t, and < 284.5 t, respectively. The lack of phosphorus and the imbalance of N/P ratio caused by quicklime disinfectants may be a factor in the changes of mangrove wetlands and surrounding nearshore waters, the growth and decline of marine species, and even global changes.

**Supplementary Information:**

The online version contains supplementary material available at 10.1038/s41598-025-02440-x.

## Introduction

Mangrove wetlands are the most ecologically valuable ecosystems on Earth, as well as one of the most severely affected and ecologically fragile natural systems^1,2^. Mangroves have a series of important ecological and socio-economic functional values, including stabilizing coastlines, wind and wave control, reducing coastal erosion, and serving as habitats for many commercially valuable invertebrate and vertebrate species^3^. Moreover, the mangrove ecosystem has high species diversity and high biological productivity due to the inclusion of all different types of animals, plants, microorganisms, and their genes^4^. Their unique characteristics make them important providers of many ecosystem services, such as breeding and nursery grounds for many commercial fish and shrimp species^5^, providing spawning, nursery, and habitat for many marine animals and rare and endangered waterfowl during seasonal migrations^6,7^. In addition, mangrove plants play an important role in the dynamic cycling of coastal sediments and nutrients, serving as important sources of atmospheric carbon dioxide fixation and coastal organic carbon^8,9^. However, in the past few decades, mangrove forests once covered over 200,000 square kilometers of tropical and subtropical coastlines, with 90% of them located in developing countries^10^. In 2020, the global mangrove forests area were 145,068 km^2^, among which Asia contained the largest coverage (39.2%). At the country level, Indonesia had the largest amount of mangrove forests, followed by Brazil and Australia^11^.

At present, "shrimp ponds (i.e. high elevation and low elevation shrimp ponds)-seawalls-tidal creek-mangrove forests" has become the main distribution pattern of mangrove coasts in China and Southeast Asia. In other words, due to the special location of mangrove wetlands at the mouth of the river in the boundary zone between land and sea, a large number of high-elevation shrimp ponds are distributed around them^12^. According to the FAO report, China, Vietnam, Ecuador, India, Indonesia, Thailand, Mexico, Bangladesh, Brazil, and the Philippines are among the top ten countries in crustacean aquaculture^13^.

**Fig. 1:**
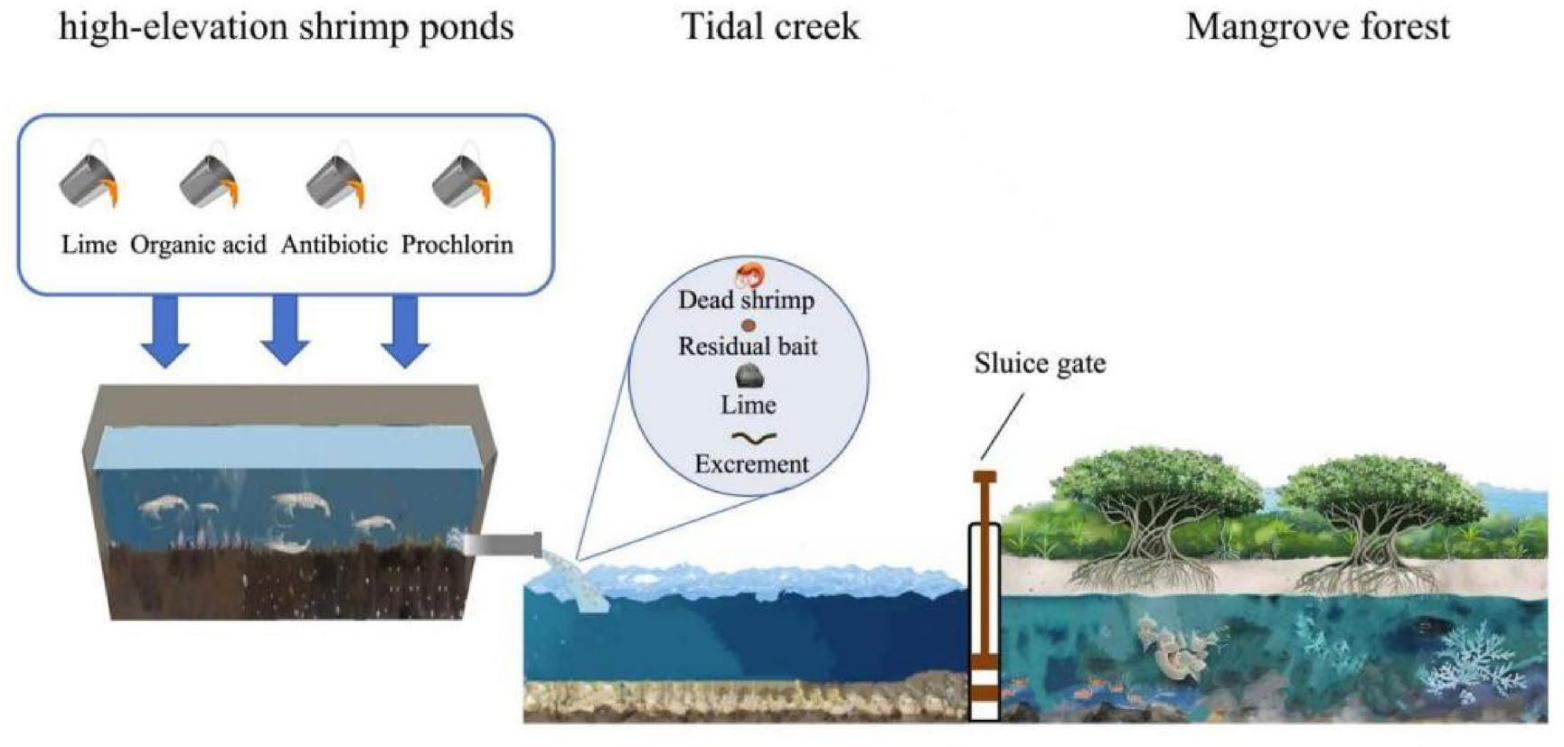
Schematic diagram of various pollutants from high-elevation shrimp ponds aquaculture discharged into mangrove ecosystem through tidal creek.

The high-elevation shrimp pond drainage system consists of a central sewage system, an outlet, and underground drainage pipes and channels (Fig. 1). The system has completely solved the problems of traditional ponds being located below sea level, which affects the dredging, disinfection, and sun drying of ponds due to incomplete drainage of shrimp ponds. At the same time, it has also improved the success rate of aquaculture. The pollution of nearshore waters caused by aquaculture and the degradation of mangrove ecosystems has become common environmental problems in tropical and subtropical coastal areas^14,15^. Therefore, in order to maintain and restore the function of mangroves, it is crucial to focus attention on the impact of chemical disinfectant emissions from high-elevation shrimp ponds on nutrients in tropical and subtropical urban estuaries and coastal areas, which have not been fully studied.

Taking the Dongzhai harbour Nature Reserve in Hainan, China as an example, there are numerous high-elevation shrimp ponds distributed around the Reserve based on the satellite image analysis (Fig. 4a). The increase in aquaculture area inevitably led to the widespread degradation of mangroves and the loss of their treatment function (Fig. S1), which in turn increased the difficulty of aquaculture. The ecological investigation results of the project team in the early stage suggested that the surface seawater of Dongzhai harbour can no longer be used for shrimp pond aquaculture due to severe degradation of mangroves. Therefore, residents started to extract groundwater for related aquaculture activities, with aquaculture wastewater was directly discharged into the mangroves through tidal creek in each shrimp culture ponds outside the protected area. As a result, the stability and living environment of the mangrove ecosystem are constantly being destroyed, leading to the continuous disappearance of mangroves^16^. Especially in East and Southeast Asia, nearly 99% of mangrove forest areas had a patch width greater than 100 m^8^.

Nitrogen and phosphorus are crucial in the growth and energy transfer processes of plants. These two elements are not only the main limiting nutrients in aquatic and terrestrial ecosystems, but also the main cause of eutrophication in water bodies. The imbalance of N/P caused by excessive levels of nutrients in water can lead to rapid proliferation of algae and other planktonic organisms, a decrease in dissolved oxygen levels, deterioration of water quality, and massive death of fish and other organisms^17,18^. The degradation of mangrove forests caused by wastewater discharge can lead to a significant increase in carbon emissions^19^. In addition, potential nitrogen mineralization processes may trigger eutrophication by increasing the bioavailability of inorganic nitrogen in mangrove soil^20^. The nitrogen-to-phosphorus ratio (N/P) in the Dongzhai harbour Nature Reserve has shown a significant upward trend during the 6-year monitoring period (Table S1). The nitrogen compounds in the form of ammonia nitrogen in the water of Dongzhai harbour are generally higher. Moreover, except for discharge outlets of pig farm and domestic sewage, the TP content in other areas was lower than the Class III surface water standard. At the same time, the monitoring results of soil available phosphorus also revealed a high degree of consistency with the phosphorus content in the water body by human input of bait, which is consistent with other studies^21^. Moreover, the plant growth at the pig farm discharge outlet was good while the species diversity was low (only *Sonneratia apetala Buch.-Ham.* and *Sonneratia caseolaris (L.) Engler*), and the benthic species such as *Cerithidea cingulata* and *Uca arcuata* were not detected. However, most of the mangrove plants at the discharge outlet of the shrimp ponds were dead and difficult to grow and survive. The previous monitoring results have shown that the pollutant emissions from high-elevation shrimp ponds were low, which the average concentration of TN (total nitrogen) and TP (total phosphorus) were 1.99 mg/L and 0.16 mg/L, respectively and the N/P ratio of the water discharged from high-level ponds and shrimp ponds is 12.4 < 16, which will not lead to an imbalance of N/P in mangrove water bodies. Nevertheless, the TP content in the water and available phosphorus content in the soil at the discharge outlet decreased significantly, which is different from other studies that shrimp farming may result in higher P contents in the adjacent soils^22,23^. Due to phosphorus being one of the important nutrients for plant growth, a lack of phosphorus can lead to the growth of phytoplankton and mangrove plants in Dongzhai Harbour, thereby affecting the biodiversity of the entire mangrove aquatic ecosystem. The emergence of this phenomenon suggested that there may be a certain connection between the reduction of phosphorus and shrimp farming behavior. Therefore, further investigation and monitoring of the entire process of high-elevation shrimp farming have been conduceted.

A pollution source investigation conducted on Dongzhai harbour found that a large amount of quicklime (CaO) was used for regular cleaning of the shrimp culture pond and increasing the alkaline reserve in the early stages of aquaculture^24^. Moreover, the production of China’s aquaculture industry currently accounts for 70% of the world’s total, and the vast majority of aquaculture uses quicklime disinfection. It is assumed that the decrease in TP content is caused by the use of quicklime because of the fact that the calcium ions generated during the disinfection process of quicklime can react with ionized phosphorus. Therefore, a calculation method for the annual emissions of wastewater, residual bait, fecal sediment, TN and TP during the aquaculture process in Dongzhai Harbor mangrove wetlands was established based on the relatively limited data provided by high-elevation shrimp pond aquaculture in the estuarine area for the mangrove ecosystem and combined with the annual use of chemical disinfectants and the total annual emissions and transformations of organic acids, to obtain the reaction amount of quicklime disinfectants with acidic phosphates and acidic substances in order to confirm that the use of quicklime disinfectants in high-elevation shrimp pond aquaculture around mangroves is the fundamental reason for the imbalance of N/P ratio in mangrove wetlands and even nearshore waters. The aim is to clarify whether the lack of phosphorus in the mangrove wetland ecosystem is related to the aquaculture of high-elevation shrimp culture ponds. Moreover, the results can also provide theoretical basis and data for the ecological protection of mangrove wetlands and even nearshore waters, especially providing reference for mangrove conservation in East Asia and Southeast Asia.

## Results and discussion

### Results of N and P ratio in water body

**Fig. 2:**
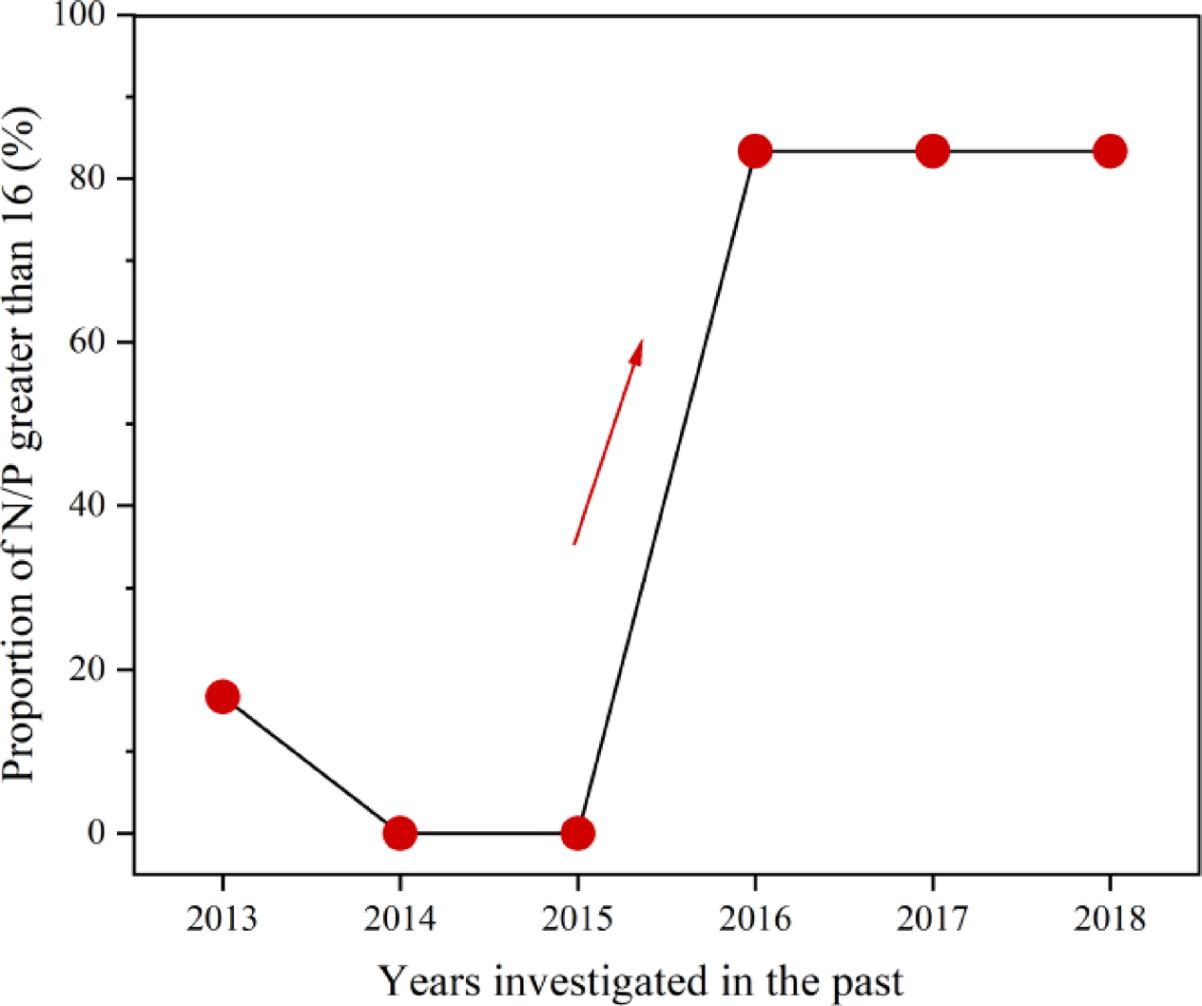
Measurement results of the proportion of N/P > 16 areas in water bodies.

It can be seen from the monitoring data in the past 6 years that the TN pollution in the water body of Dongzhai Harbor was relatively serious, while the total phosphorus pollution problem is not significant, with it’s concentration was mostly < 0.1 mg/L during most periods. The measurement results of the sampling points and area in Fig. 4 showed that the correlation between TN and TP was not significant, with the lowest TN/TP ratio being 3.46 and the highest being 58.63, indicating a significant difference (Table S1). Among them, the regions with N/P > 16 were particularly prominent in the C and E regions. The demand for N and P by phytoplankton indicates that normal growth can be achieved when the N/P ratio of the water is maintained at 16:1. If the N/P ratio is greater than 16, the water is in a phosphorus deficient state. From the perspective of the relative evaluation rule of nutrient limitation, the conclusion can be drawn that P may be prioritized for utilization and one of the limiting factors for the growth of phytoplankton and mangrove plants in Dongzhai Harbour. Lack of phosphorus can lead to restricted growth of aquatic plants, thereby affecting the biodiversity of the entire mangrove aquatic ecosystem. It is worth noting that the shrimp farming was stopped because of the outbreak of the *sphaeromadae* in 2012 and restored in 2016 with the insect attack was eliminated. According to monitoring data analysis, more than 80% of the areas in Dongzhai Harbor have N/P values greater than 16 from 2016 to 2018 (Fig. 2).

The average value of SS, TN, and TP from discharge of the high-elevation shrimp pond in Dongzhai harbour was 82.8 mg/L, 2.0 mg/L, and 0.16 mg/L, respectively. Dongzhai harbour is a collection area for 1866.8 hectares of high-elevation shrimp pond wastewater. A large amount of shrimp pond aquaculture wastewater flows through tidal creek, Yanfeng East River, Yanfeng West River and other channels in the Dongzhai harbour Mangrove Nature Reserve, and finally flows into Puqian Bay. If a 1500 hectares shrimp pond is in normal use (80% utilization rate), with an average water depth of 2.5 m, a daily average drainage of 2.3%, an average cultivation period of 120 days, and an annual average production of 2.3 cycles, the volume of the high-elevation shrimp culture ponds can be obtained as: V = 1500 ha × 10,000 m^2^/ha × 2.5 m = 3.8 × 10^7^ m^3^. Moreover, the annual drainage of shrimp ponds was: Q = 37,500,000 m^3^ × 0.023 × 120 × 2.3 = 2.4 × 10^8^ m^3^/a. If the daily amount of sewage flowing through the protected area is about 652,300 cubic meters, the annual discharge of residual bait and fecal sediment can reach an astonishing 2.0 × 10^4^ t, with TN and TP of 473.7 t and 38.1 t, respectively.

### Results of N and P pollution emissions

#### Analysis of Ca(OH)_2_ emission from quicklime

Due to the fact that the characteristic pollutants emitted during the different stages of shrimp farming were also different, and some of these pollutants can react with each other in tidal creek, causing the inability to measure the true content of Ca(OH)_2_ produced after lime disinfection. The complexity of this system led to inaccurate on-site sampling and monitoring results, therefore, it is necessary to use laboratory simulation of the use of chemical disinfectants.

The disinfection principle of quicklime pond cleaning is that quicklime generates strong alkaline calcium hydroxide and releases a large amount of heat when it comes into contact with water^25^. Strong alkali can quickly achieve sterilization and disinfection at high temperatures. At the same time, calcium hydroxide exposed to air will generate calcium carbonate, which can soften the sediment and improve its ventilation conditions, leading to faster and more complete decomposition of organic matter in the sediment. The reaction is as follows:

1 $$CaO+{H}_{2}O\to Ca(OH{)}_{2}$$

2 $$Ca(OH{)}_{2}+C{O}_{2}\to CaC{O}_{3}+{H}_{2}O$$

In the simulated shrimp pond experiment in the laboratory, after the water body had stabilized and 48 h later, the middle layer was taken and the concentration of Ca(OH)_2_ were measured to be 1.7 g/L and 1.4 g/L, respectively. Since a solubility of 0.3 g/L of Ca(OH)_2_, which was converted into a solid CaCO_3_ of 0.4 g/L, the calculated attenuation failure rate of saturated calcium hydroxide solution was 0.005 h^−1^.

According to the simulation experiment using 1500 kg of quicklime per hectare during pond cleaning, the amount of Ca(OH)_2_ in the turbid liquid discharged from 1 hectare of shrimp pond was 379.3 kg, and the amount of solid Ca(OH)_2_ was 1379.3 kg. In addition, 298.5 kg of CaCO_3_ solid waste was discharged into the surrounding environment of the shrimp pond. It should be pointed out that the concentration of Ca(OH)_2_ in the discharged water from the pond was as high as 1.8 g/L, which exceeded the saturation concentration of Ca(OH)_2_, indicating that insoluble Ca(OH)_2_ was discharged into the mangrove wetland during the release of the pond wastewater, with the pH of the wastewater was correspondingly 12.8. Assuming that all shrimp culture ponds are cleaned with quicklime simultaneously, it can be calculated that a single cleaning activity of the shrimp culture pond can discharge alkaline wastewater containing Ca(OH)_2_ (1866.8 × 80% × 379.3 = 566.7 tons), CaCO_3_ (1866.8 × 80% × 298.5 = 445.8 tons), and Ca(OH)_2_ solid waste (1866.8 × 80% × 1379.3 = 2060.8 tons) into the environment with the use of quicklime disinfectant. As large areas of shrimp culture ponds are disinfected with quicklime at the same time, a large amount of alkaline pollutants will be discharged, resulting in instantaneous strong alkalinity in the water environment. The annual discharge of soluble Ca(OH)_2_ and solid Ca(OH)_2_ in the shrimp pond of Dongzhai harbour is 1303.4 t/a and 4739.8 t/a based on the annual aquaculture cleaning of 2.3 times in the Hainan region, respectively. Accordingly, the total annual discharge of Ca(OH)_2_ is as high as 6043.2 t/a.

#### Analysis of Ca(OH)_2_ reactivity

Large scale discharge of wastewater containing Ca(OH)_2_ can alkalize the drained originally acidic sulfate soil of the mangrove forest in Dongzhai harbour^26^, which was consistent with the monitoring results of previous finding that the soil in certain areas of Dongzhai harbour was slightly alkaline. At the same time, the solid organic matter discharged from shrimp pond aquaculture also underwent acidification reactions when deposited into the sediment of mangroves^27^. The estimated daily total amount of organic acids discharged into the mangrove wetland protection area during shrimp pond aquaculture was: W = 97.3 kg/d + 52.9 kg/d ~ 84.7 kg/d + 2.1 × 10^3^ kg/d + 4.8 × 10^3^ ~ 5.6 × 10^3^ kg/d = 7.1 × 10^3^ kg/d ~ 7.8 × 10^3^ kg/d, i.e 2,576.9 ~ 2,858.0 t/a.

1 mol of Ca(OH)_2_ requires 2 mol of RCOOH according to the Eq. (9). Assuming that the organic acid is the acetic acid produced most during anaerobic fermentation process (75%), 74 g of Ca(OH)_2_ requires 120 g of CH_3_COOH. The rate limiting factor for the reduction of organic acids mainly depends on the diffusion rate of Ca(OH)_2_ in the sediment based on the previous study^28^, it has been calculated that the permeation rate of Ca(OH)_2_ molecules is about 2.78 cm/d. The organic acids in the 0–10 cm soil were completely neutralized on the 4th day, while the organic acids on the 18th day. Therefore, if methane anaerobic conversion is carried out through the acetic acid pathway within 15 days, 1.0 t of organic acid is completely reacted by calcium hydroxide, which will consume 0.6 t of Ca(OH)_2_ at 90% conversion, the remaining 10% organic acid consumes 154.6–171.5 t of Ca(OH)_2_ annually.

The results of laboratory simulation calculations showed that the effective chlorine produced trichloroisocyanuric acid was 1.0 t/cycle, and 1 mol Ca(OH)_2_ required the consumption of 2 mol HClO, which meant 74 g Ca (OH)_2_ required the consumption of 105 g HClO. 1.0 t of available chlorine was completely reacted with only 0.7 t of Ca(OH)_2_, leaving 566.0 t of calcium hydroxide. Therefore, the amount of Ca(OH)_2_ consumed by the neutralization reaction of the disinfectant in the high position shrimp pond aquaculture was very small, with an annual consumption of 1.7 tons. In addition, the seawater carbon dioxide system is composed of carbon dioxide, carbonic acid, bicarbonate ions, and carbonate ions dissolved in seawater^29^. The calcium ions and carbonate ions in seawater can form calcium carbonate precipitation, which means that the Ca(OH)_2_ emitted from high-elevation shrimp culture ponds will also be neutralized and consumed by the dissolved CO_2_ in seawater. The precipitation rate of calcium carbonate is not only related to saturation, but also to the presence of organic matter, phosphorus compounds, and magnesium ions in seawater.

Furthermore, calcium ions (Ca^2+^) in water can form slightly soluble phosphorus precipitates with phosphorus ions (PO_4_^3−^), hydrogen phosphorus ions (HPO_4_^2−^), and dihydrogen phosphorus ions (H_2_PO_4_^2−^) in the water, including HAP (Ca_5_(PO_4_)_3_(OH)), OCP (Ca_8_H_2_(PO_4_)_6_·5H_2_O), DCPD (CaHPO_4_·2H_2_O) and TCP (Ca_3_(PO_4_)_2_·nH_2_O). According to calculations, the annual discharge of TP from livestock, poultry breeding wastewater and domestic sewage in Dongzhai Harbour is 10.0 t/a and 63.0 t/a, respectively. Adding the TP discharged from shrimp ponds (38.1 t/a), the total annual discharge of TP is over 111.1 t/a. However, the phosphorus concentration was not increased. It can be inferred that the amount of Ca(OH)_2_ completely consumed by TP (calculated as orthophosphorus) per year should be below 284.5 tons. The monitoring of organic phosphorus in water bodies is somewhat challenging, and the composition of marine systems is complex. Therefore, the amount of calcium phosphorus that Ca(OH)_2_ can form is also a topic that requires further research according to the consumption situation of Ca(OH)_2_.

**Fig. 3:**
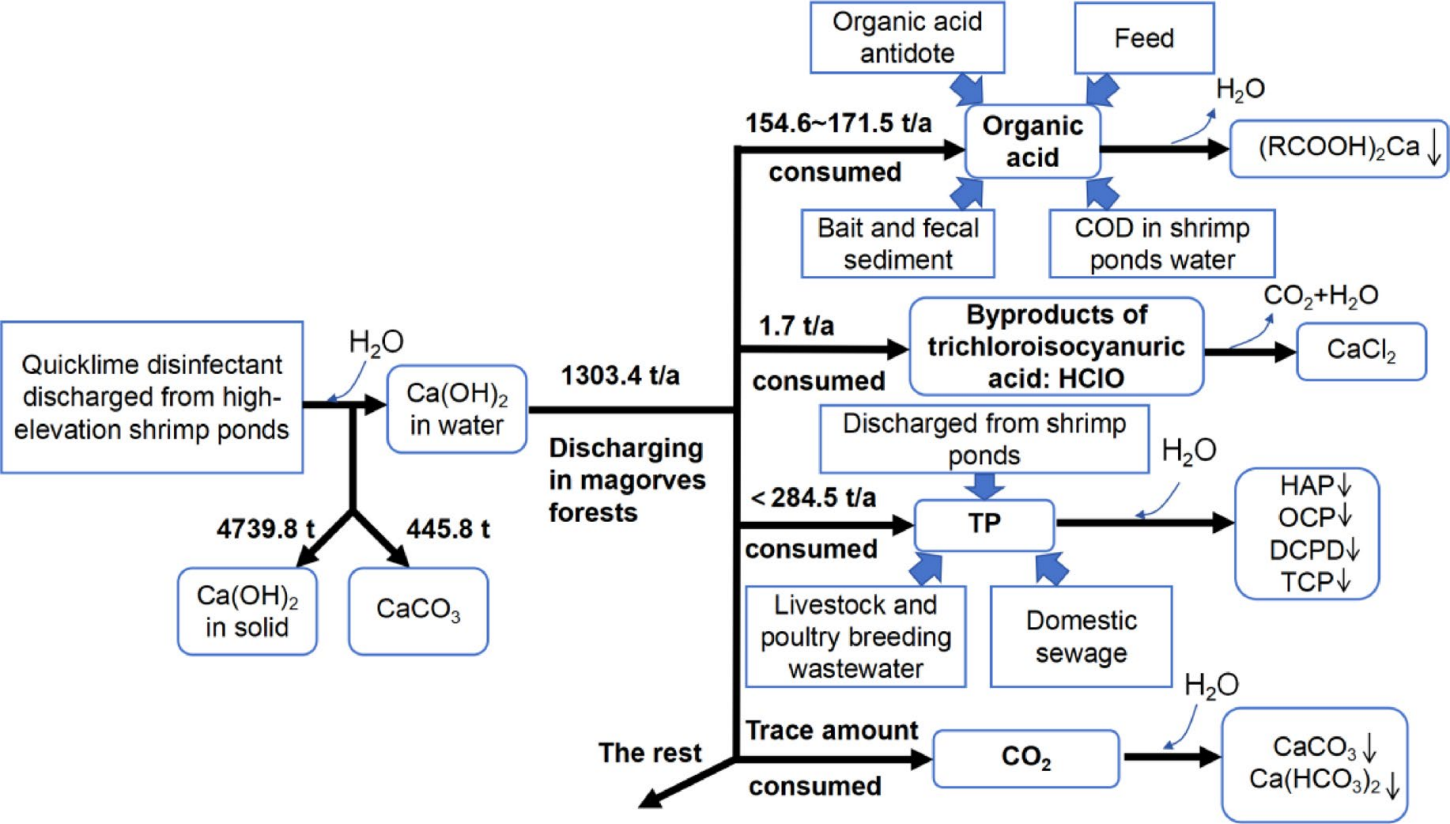
Reaction quantity and transformation process of Ca(OH)_2_ in Dongzhai Harbor Ecosystem.

The reaction amount and transformation process of Ca(OH)_2_ in the Dongzhai Harbor ecosystem were shown in Fig. 3, which is an ideal reaction process under uniform conditions. However, the marine system is complex and greatly affected by tides, so the complete reaction between TP and Ca(OH)_2_ was only evident at the shrimp pond discharge outlet. In summary, the use of quicklime disinfectants is one of the main reasons for the low TP content in water. The weakly alkaline water in Dongzhai harbour promoted the formation of calcium phosphorus precipitation, causing TP to become a limiting factor in the mangrove ecosystem of Dongzhai harbour. Moreover, the fixation effect of calcium phosphate will be very strong when the calcium content in water is high, thereby reducing the efficiency of plant utilization of phosphorus^30–32^. The reduction of phosphorus may be the main reason for maintaining a certain degree of stability in the ecosystem of Dongzhai harbour, which is consistent with research findings in other aquaculture areas such as Laizhou Bay in the Bohai Sea^33,34^ and Sanggou Bay in the Yellow Sea^35^. The phosphate loss caused by the input of calcium hydroxide requires a longer period of time, and coupled with the longer shrimp farming time in China, therefore, the phenomenon of nitrogen phosphorus imbalance is more pronounced compared to other countries and regions. The decrease of TP content led to N/P > 16 through the use of quicklime disinfectant, in water ecosystems based on phosphorus geochemical cycles, phosphorus becomes a limiting factor due to its inability to meet the needs of primary producers. On the other hand, the imbalance of N/P ratio caused by phosphorus deficiency in water bodies may affect the ecological environment of mangrove wetlands and surrounding nearshore waters. Such influence may even be related to the succession of marine species and global ecological environment changes.

## Conclusion

Coastal nutrient pollution has been identified as one of the biggest threats facing these valuable systems worldwide. Mangrove wetlands and their nearshore waters are most susceptible to the anthropogenic pressure of releasing quicklime disinfectants from high-elevation shrimp ponds, but this impact is severely underestimated. The reaction process and amount of Ca(OH)_2_ emissions in mangrove ecosystems has been shown on the basis of establishing the annual emission of pollutants during the aquaculture process in high-elevation shrimp ponds. According to the research results, it is first proposed that the lack of phosphorus in mangrove wetland ecosystems and nearshore waters is related to high-elevation pond aquaculture. The Ca(OH)_2_ generated during the disinfection process can react with soluble inorganic phosphorus in the water to form stable calcium phosphorus precipitates, resulting in a decrease in TP content in the water and an imbalance in the N/P ratio. In other words, the quicklime disinfectant used in high-elevation shrimp farming is the fundamental cause of the imbalance in N/P ratio in mangrove wetlands, nearshore seas, or water bodies. The variation in phosphorus content may be a factor causing changes in the ecological environment of mangrove wetlands and even surrounding nearshore waters, which is a brand new topic worth exploring.

Moreover, a new approach to pond cleaning should be implemented to prevent environmental damage caused by shrimp farming in the future breeding process, for example, minimizing the use of quicklime in clearing ponds through scientific practice. Otherwise, excessive liming can lead to high costs and overly alkaline soil, which may cause various issues, such as nutrient imbalances, increased susceptibility to certain diseases, and environmental harm to surrounding areas. Furthermore, considering that alkaline lime water and effective chlorine acid disinfectants can undergo acid–base neutralization reactions, it is possible to implement zoning management of high-elevation shrimp ponds around Dongzhai Harbour based on the distribution of sewage outlets, and staggered pond cleaning and aquaculture should be conducted for each area’s shrimp ponds. Some shrimp ponds should use quicklime to clean the pond, while others can use acidic disinfectants to neutralize the acidic and alkaline pollutants discharged in the tidal ditch upstream of the discharge outlet, reducing the concentration of pollution emissions from the discharge outlet.

## Material and methods

### Description of study area

#### Study area

The high-elevation shrimp ponds drainage system consists of a central sewage system, an outlet, underground drainage pipes and channels. The high-elevation shrimp aquaculture system has completely solved the problems of traditional ponds being located below sea level, which affects the dredging, disinfection, and sun drying due to incomplete drainage. Moreover, it has also improved the success rate of aquaculture.

**Fig. 4:**
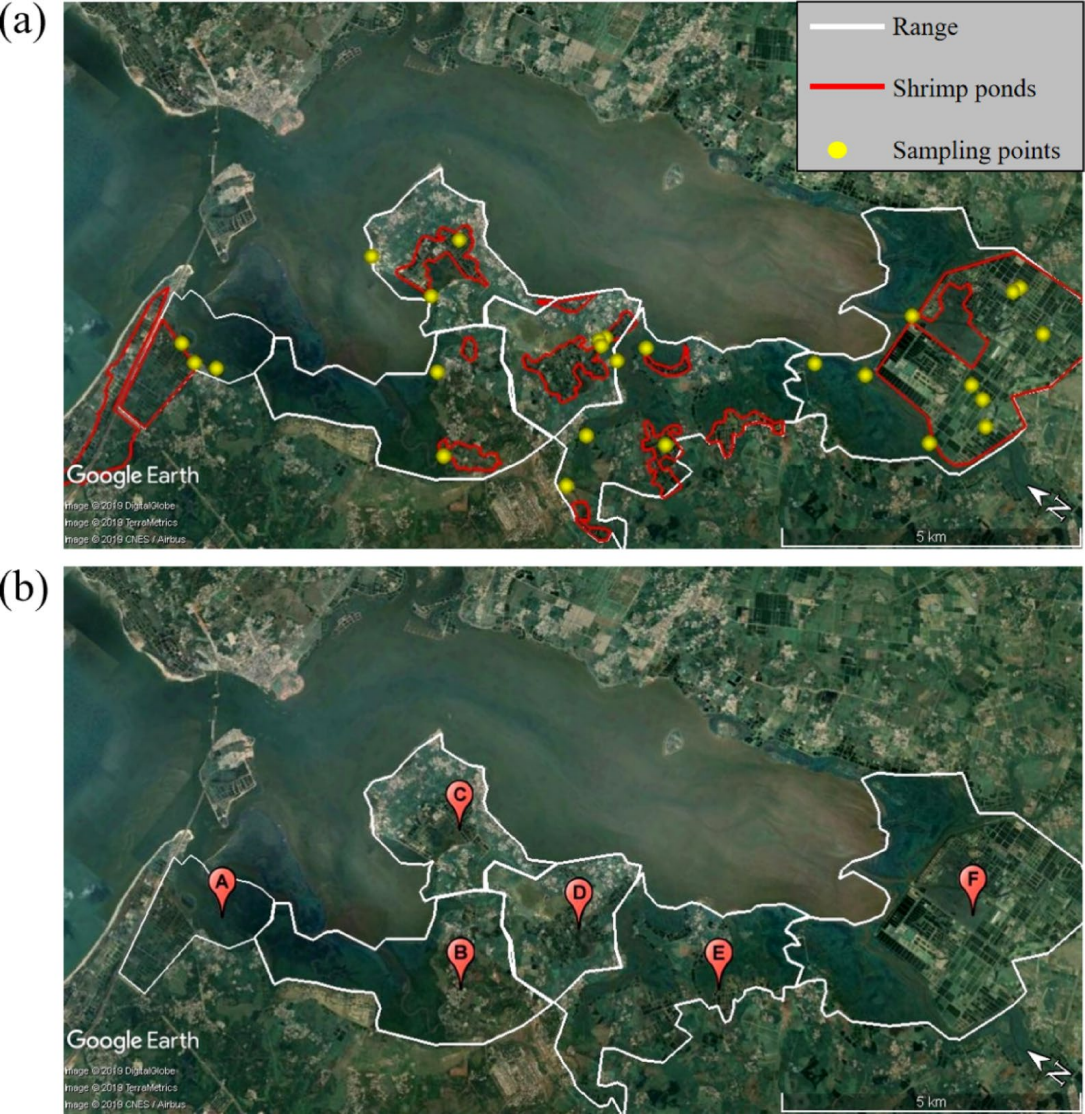
Image of study area created by Google Earth Pro (7.3.6.10201): (**a**) Boundary map of Dongzhai Harbor Conservation Area and Shrimp Pond Culture Area; (**b**) Division of sampling area (A: Tashi; B: Yanzhong Country; C: Shilu Country; D: Shanwei Country; E: Yanfeng; F: Sanjiang).

The Dongzhai Harbour Mangrove National Nature Reserve in Hainan is the largest mangrove conservation area in China, located at longitude 110° 32′–110° 37′ east and latitude 19° 51′–20° 1′ north. The protected area covers 3337.6 hectares with a mangrove forest area of 2065 hectares^36^. It is shown that there are approximately 1866.8 hectares of shrimp ponds around Dongzhai Harbor Nature Reserve, mainly high-elevation ponds based on the analysis of satellite images and field investigations. The main distribution areas of shrimp ponds are Ten thousand acres-shrimp ponds in Sanjiang Town, Tashi and Yanfeng Town (Fig. 4b and Table S2), with the sampling point setting method can be seen in Text S1.

#### Calculation for pollution emissions from high-elevation shrimp culture ponds of Dongzhai harbour

The annual discharge amount of wastewater, total solids (TDS), total phosphorus (TP), total nitrogen (TN), and biochemical oxygen demand (BOD) of the main pollutants discharged from the shrimp ponds in the vicinity of Dongzhai harbour Mangrove Wetland were calculated by acquiring the concentration of the nutrients and the volume of the discharged wastewater based on the pollution monitoring results in the past 6 years, satellite image analysis and field investigation results.

The calculation formula for shrimp pond volume (V) is as follows:

3 $${\text{V }}\left( {{\text{m}}^{{3}} } \right) = {\text{H}} \times {\text{M}}$$

Which H and M represent the average depth (m) and area (m^2^) of shrimp pond, respectively.

Annual discharge of shrimp pond (Q) and annual emissions of pollutants (Q′) can be determined through the following eqation:

4 $${\text{Q}} = {\text{V}} \times \eta \times {\text{D}} \times {\text{T}}$$

5 $${\text{Q}}^{\prime } = {\text{Q}} \times {\text{C}}_{{\text{m}}}$$

which ƞ, D, and T represent the average daily drainage, the number of breeding days per cycle and the average annual breeding cycle., respectively, and C_m_ represent the concentration of the pollutants (TN, TP and SS, mg/L) determined by standard methods (HJ636-2012, HJ 671-2013, GB11901-89)^37^.

### Simulation and calculation methods for the use process of quicklime disinfectants

#### Simulation method

**Fig. 5:**
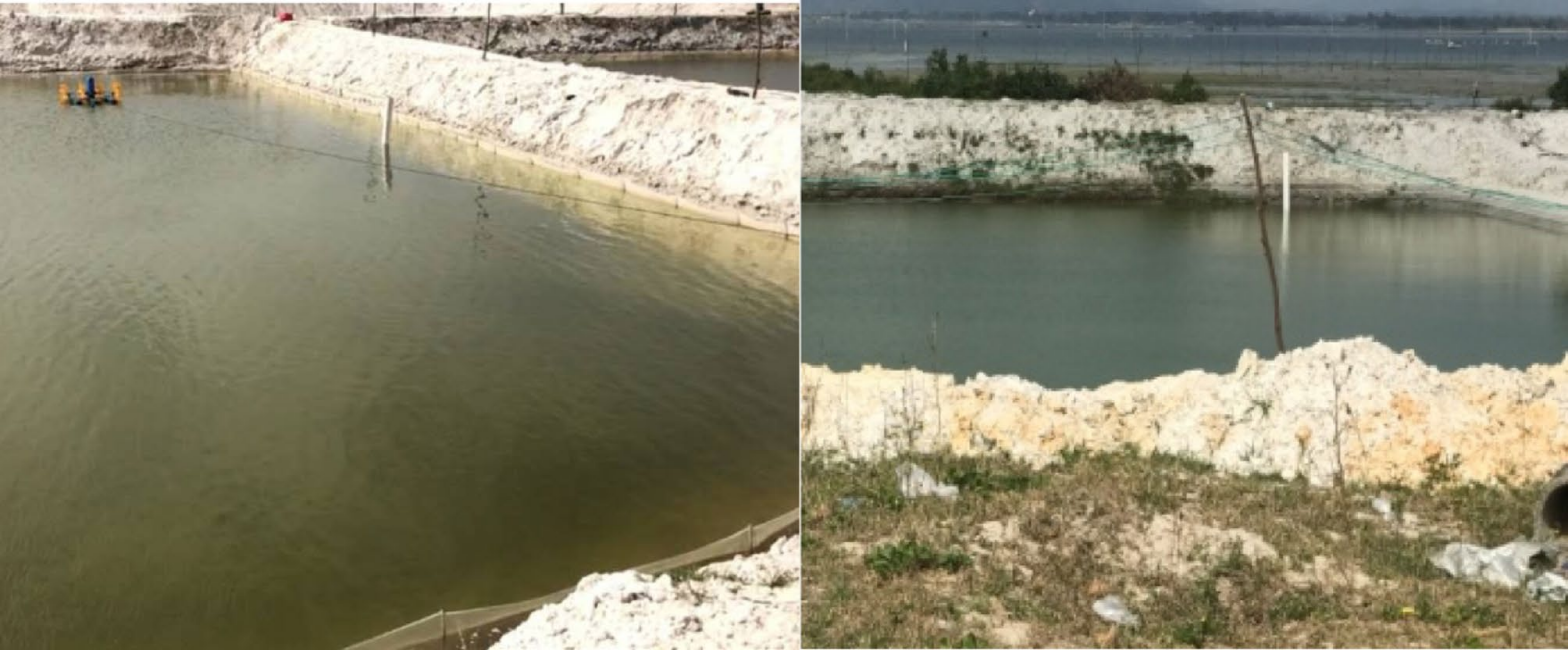
Discharge diagram using the solid alkaline disinfectant quicklime around the shrimp pond.

The disinfection principle of quicklime pond cleaning is that quicklime reacts with water can generate strong alkaline Ca(OH)_2_ and releases a large amount of heat, which can quickly achieve the effect of sterilization and disinfection. According to data, the vast majority of farmers choose quicklime for cleaning with sediment during the cleaning process of shrimp farming. The amount of quicklime used during pond cleaning is large, with a dosage of 100 kg per acre. After the pond cleaning is completed, all the pond water and sediment at the bottom of the pond need to be discharged (Fig. 5).

If soil is used for simulation experiments, all components in the soil will mix with solid Ca(OH)_2_ and harden together, making it impossible to determine the amount of solid Ca(OH)_2_ generated using the gravimetric method. Moreover, some compounds in the soil can make it difficult to accurately determine the concentration of soluble Ca(OH)_2_ in clarified lime water, the amount of solid Ca(OH)_2_ and the emissions of CaCO_3_ solid waste. To eliminate the interference caused by various factors in the soil, the method of simply dissolving quicklime in distilled water to calculate the production of soluble Ca(OH)_2_ and solid Ca(OH)_2_ was used in this experiment, with the specific plan is as follows:

Under temperature conditions of 25 ± 2 °C, three rectangular plastic boxes (54 cm × 45 cm × 35 cm) were selected as simulated high-elevation shrimp culture ponds, with one as a blank control and the other two as parallel experiments.

1. Experimental group: (i) 36.4 g of lime was evenly sprinkled at the bottom of the box (100 kg of quicklime was required for every 667 m^2^); (ii) 5 L of deionized water was added according to the actual application ratio to immerse lime and let it mature; (iii) After mixing the lime evenly the next day, the water was drained after soaking for 2 days; the pH of drainage, the concentration of Ca(OH)_2_ in clarified lime water and the content of Ca(OH)_2_ in discharged solid waste were measured.
2. Control group: 5 L of deionized water was added directly to the empty box and stirred the next day. The pH and the concentration of Ca(OH)_2_ of the water were measured after settling for 2 days.

#### Simulation method for calculating the attenuation coefficient of quicklime

This study took the complete loss of alkalinity of quicklime as the criterion for failure, including the attenuation of alkaline wastewater and alkaline solid waste generated by quicklime disinfection. Ca(OH)_2_ in water mainly failed through acid–base reactions and its combination with carbon dioxide in the air. In this study, due to the absence of acid–base reactions during lime pond cleaning, only the failure rate of its combination with carbon dioxide in the air was measured.

Calculation of Alkaline Lime Water Attenuation: In the operation aforementioned, the middle layer of the control group and the experimental group was taken every 24 h to measure the concentration of Ca(OH)_2_. The attenuation coefficient and solid content of saturated calcium hydroxide solution can be calculated by the difference between two measurements.

#### Calculation for the amount of quicklime discharged from the shrimp ponds in the vicinity of Dongzhai harbour

The calculation formula for the amount of Ca(OH)_2_, CaCO_3_, and Ca (OH)_2_ solid waste discharged during a pond cleaning process are as follows:

6 $${\text{CCa}}\left( {{\text{OH}}} \right)_{{2}} \left( {{\text{kg}}} \right) \, = {\text{M}} \times {8}0\% \times {\text{C}}^{\prime } {\text{Ca}}\left( {{\text{OH}}} \right)_{{2}}$$

7 $${\text{CCaCO}}_{{3}} \left( {{\text{kg}}} \right) = {\text{M}} \times {8}0\% \times {\text{C}}^{\prime } {\text{CaCO}}_{{3}}$$

8 $${\text{CCa}}\left( {{\text{OH}}} \right)_{{2}} - {\text{solid}}\left( {{\text{kg}}} \right) = {\text{M}} \times {8}0\% \times {\text{C}}^{{\prime }} {\text{Ca}}\left( {{\text{OH}}} \right)_{{2}} - {\text{solid}}$$

Which M, 80% and C′ represent the shrimp pond area (ha), shrimp pond utilization rate, and the amount of alkaline pollutants generated per hectare of shrimp pond during disinfection process (kg), respectively.

Estimation Method of Ca(OH)_2_ Reaction Quantity in Dongzhai Harbour Ecosystem

The use of quicklime disinfectants led to the discharge of a large amount of strong alkaline disinfection (dissolved Ca(OH)_2_) into the mangrove ecosystem. The composition of the marine system is complex, and the chemicals that can react with Ca(OH)_2_ include acidic compounds in the mangrove water such as organic acids used in shrimp pond aquaculture, weak acidic disinfectants (the main byproduct is HClO), acidic phosphorus compounds, and CO_2_ in seawater. According to their reaction equation with calcium hydroxide, the reaction amount of Ca(OH)_2_ under different pathways can be obtained, as shown in the following equation:

9 $$Ca\left( {OH} \right)_{2} + 2RCOOH \to (RCOOH)_{2} Ca \downarrow + 2H_{2} O$$

10 $$Ca\left( {OH} \right)_{2} + 2HClO \to CaCl_{2} + 2H_{2} O + O_{2} \uparrow$$

11 $$5Ca^{2 + } + 3PO_{4}^{3 - } + OH \to Ca_{5} (PO_{4} )_{3} \left( {OH} \right) \downarrow$$

12 $$8Ca^{2 + } + 6PO_{4}^{3 - } + 2H^{ + } + 5H_{2} 0 \to Ca_{8} H_{2} (PO_{4} )_{6} \cdot 5H_{2} O \downarrow$$

13 $$Ca^{2 + } + HPO_{4}^{2 - } + 2H_{2} O \to CaHPO_{4} \cdot 2H_{2} O \downarrow$$

14 $$3Ca^{2 + } + 2PO_{4}^{3 - } + nH_{2} O \to Ca_{3} (PO_{4} )_{2} \cdot nH_{2} O \downarrow$$

The calculation formula for the daily estimated total organic acid emissions (W,) into the mangrove wetland protection area during shrimp pond aquaculture is as follows:

15 $${\text{W }}\left( {{\text{kg}}/{\text{d}}} \right) = {\text{W}}_{{1}} + {\text{W}}_{{2}} + {\text{W}}_{{3}} + {\text{W}}_{{4}}$$

Which W_1_, W_2_, W_3_, W_4_ represent the amount of organic acid antidote emissions, the amount of organic acids conversed in feed, fecal sediment and COD in shrimp pond water under anaerobic conditions (kg/d).

In addition, laboratory experiments have shown that Ca (OH)_2_ can react with CO_2_ in the atmosphere at a decay rate of 0.005/h, but the amount generated is low.

## Electronic supplementary material

Below is the link to the electronic supplementary material.

Supplementary Material 1

## Data Availability

All data supporting the finding of this study are available within this article and Supplementary Data.
