# Supplementary material for: Analysis of the causes of N/P imbalance in mangrove water caused by high elevation shrimp ponds
Source: Sci Rep. 2025 May 20;15:17424. doi: 10.1038/s41598-025-02440-x (PMC12092609; doi:10.1038/s41598-025-02440-x)
Supplement: Supplementary file 1 — Supplementary Material 1 [file 41598_2025_2440_MOESM1_ESM.docx]

**Supplementary Data**

**Title:**

**Analysis of the causes of N/P imbalance in mangrove water caused by high elevation shrimp ponds.**

**Authors:**

Yunan Yang ^1 *^, Zhe Li ^1^, Nan Zhou ^1^, Yangang Lin ^1^, Qian Sheng ^1^, Myat Thiri ^1,2^, Yao Wang ^1^

1 School of Space and Environment, Beihang University , Beijing 100191 , China

2.Biotechnology Research Department, Ministry of Education, Kyauk Se Township, Mandalay Division, 15011, Republic of Union of Myanmar

* Corresponding author, E-mail: [yangyn@buaa.edu.cn](mailto:yangyn@buaa.edu.cn)

**List (1 Text, 2 Tables, 1 Figure):**

**Text S1 *Sampling frequency and sampling point settings***

Based on the tidal periodicity of seawater in Dongzhai Harbor Mangrove Reserve, the sampling frequency was set to two quarters: rainy and dry seasons to ensure continuous and periodic sampling. The rainy season from May to November; Dry season from December to April of the following year.

The sampling points were divided into 6 different areas based on the distribution of mangrove growth areas, shrimp ponds, and human activities, to collect water (high tide, low tide) and soil samples. Due to the low tide being the discharge period of the shrimp pond, the various indicators measured in the water should be able to more accurately reflect the water pollution status of the mangrove forest. Therefore, samples were collected during the high and low tide periods at the same point. Due to the further serious trend of pollution in Dongzhai Harbor after 2016, the monitoring of pollution sources in surrounding shrimp ponds has been strengthened, and the sampling points have increased significantly.

**Table S1 The ratio of TN to TP (N/P) in the sampling area**

| **Sampling area** | **Time** | **TN/TP** |
| --- | --- | --- |
| Tashi | 2013 rainy season | 5.65 |
|  | 2014 dry season | 11.80 |
|  | 2014 rainy season | 5.43 |
|  | 2015 dry season | 8.12 |
|  | 2016 rainy season | 10.24 |
|  | 2017 dry season | 46.89 |
|  | 2018 rainy season | 26.00 |
|  | 2018 dry season | 24.29 |
| Yanzhong Country | 2013 rainy season | 11.89 |
|  | 2014 dry season | 12.00 |
|  | 2014 rainy season | 5.10 |
|  | 2015 dry season | 3.46 |
|  | 2016 rainy season | 23.92 |
|  | 2017 dry season | 58.63 |
|  | 2018 rainy season | 7.00 |
|  | 2018 dry season | 17.33 |
| Shilu Country | 2013 rainy season | 55.50 |
|  | 2014 dry season | 4.75 |
|  | 2014 rainy season | 15.50 |
|  | 2015 dry season | 8.22 |
|  | 2016 rainy season | 19.25 |
|  | 2017 dry season | 36.08 |
|  | 2018 rainy season | 23.00 |
|  | 2018 dry season | 58.00 |
| Shanwei Country | 2013 rainy season | 12.78 |
|  | 2014 dry season | 10.29 |
|  | 2014 rainy season | 11.58 |
|  | 2015 dry season | 5.35 |
|  | 2016 rainy season | 34.22 |
|  | 2017 dry season | 49.50 |
|  | 2018 rainy season | 12.00 |
|  | 2018 dry season | 22.00 |
| Yanfeng | 2013 rainy season | 5.89 |
|  | 2014 dry season | 11.44 |
|  | 2014 rainy season | 13.64 |
|  | 2015 dry season | 1.26 |
|  | 2016 rainy season | 28.11 |
|  | 2017 dry season | 57.50 |
|  | 2018 rainy season | 24.40 |
|  | 2018 dry season | 39.67 |
| Sanjiang | 2013 rainy season | 9.00 |
|  | 2014 dry season | 12.6 |
|  | 2014 rainy season | 11.20 |
|  | 2015 dry season | 3.50 |
|  | 2016 rainy season | 35.00 |
|  | 2018 rainy season | 20.43 |
|  | 2018 dry season | 35.00 |

**Table S2 Geographic coordinates of sample collection point**

| **Sample collection point** | **Geographic coordinates** |
| --- | --- |
| Tashi | 20°0'N，110°32'E |
| Tashi 2 | 20°0'N，110°32'E |
| Tashi 3 | 20°0'N，110°32'E |
| Shilu Country 1 | 19°59'N，110°34'E |
| Houpai Country | 19°58'N，110°33'E |
| Houpai Country 2 | 19°58'N，110°32'E |
| oceanic trench | 19°56'N，110°34'E |
| Shrimp pond | 19°57'N，110°34'E |
| Sanjiang Gate | 19°55'N，110°37'E |
| Mud sampling point | 19°54'N，110°36'E |
| Mixed breeding area | 19°54'N，110°36'E |
| Fish pond in ten-thousand shrimp ponds | 19°54'N，110°36'E |
| Shrimp pond in ten-thousand shrimp ponds | 19°54'N，110°36'E |
| East Trench in ten-thousand shrimp ponds | 19°54'N，110°37'E |
| Shrimp pond 2 in the east of ten-thousand shrimp ponds | 19°54'N，110°37'E |
| Shrimp pond 3 in the east of ten-thousand shrimp ponds | 19°54'N，110°37'E |
| Sanjiang Discharge outlet | 20°0'N，110°32'E |
| Xitou Country | 20°0'N，110°32'E |
| Reserve wharf 2 | 19°57'N，110°34'E |
| Left side discharge outlet of the protected area | 19°57'N，110°34'E |
| Medium side discharge outlet of the protected area | 19°57'N，110°35'E |
| Right side discharge outlet of the protected area | 19°57'N，110°35'E |
| Discharge outlet of Niuluo mountain in Yanfeng | 19°56′N，110°33′E |
| Daoxue natural forests | 19°55′N，110°36′E |


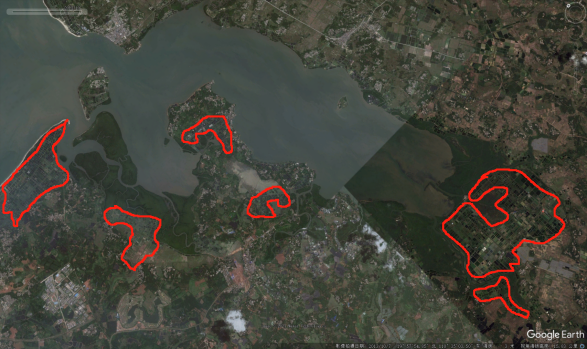

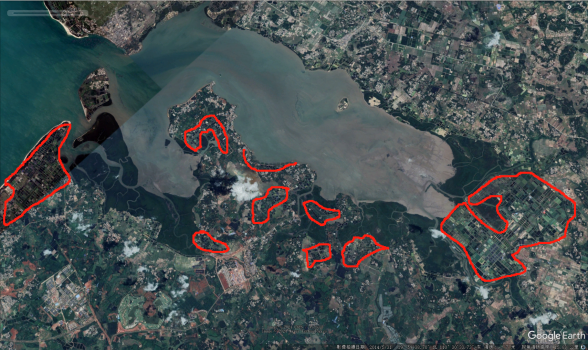


2014

2013


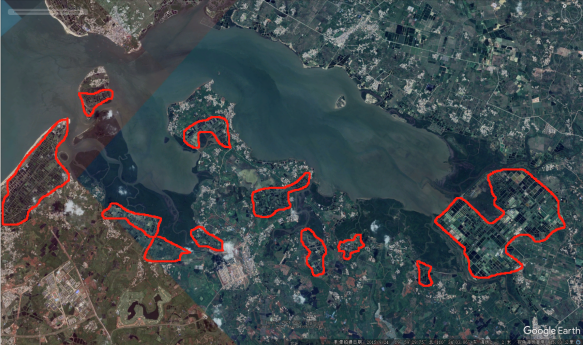

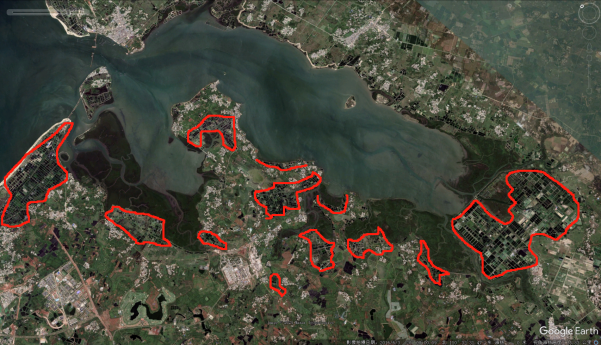


2016

2015


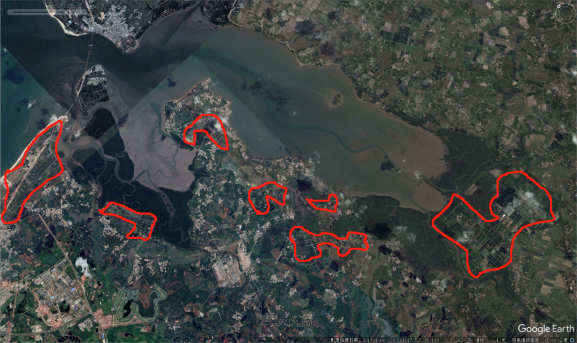

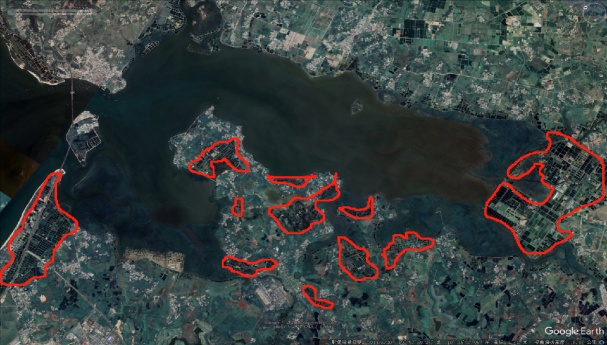


2017

2018

**Fig. S1** Variations in the high-elevation shrimp pond aquaculture area of Dongzhai Harbor from 2013 to 2018, with the red line represents the boundary of shrimp ponds.
